# Characterization of a Novel Mouse Model of Multiple Myeloma and Its Use in Preclinical Therapeutic Assessment

**DOI:** 10.1371/journal.pone.0057641

**Published:** 2013-02-21

**Authors:** Rosemary A. Fryer, Timothy J. Graham, Emma M. Smith, Simon Walker-Samuel, Gareth J. Morgan, Simon P. Robinson, Faith E. Davies

**Affiliations:** 1 Haemato-Oncology Research Unit, The Institute of Cancer Research, London, United Kingdom; 2 Cancer Research UK & EPSRC Cancer Imaging Centre, The Institute of Cancer Research, London, United Kingdom; 3 Centre for Advanced Biomedical Imaging, University College London, London, United Kingdom; Istituto di Ricerche Farmacologiche Mario Negri, Italy

## Abstract

To aid preclinical development of novel therapeutics for myeloma, an *in vivo* model which recapitulates the human condition is required. An important feature of such a model is the interaction of myeloma cells with the bone marrow microenvironment, as this interaction modulates tumour activity and protects against drug-induced apoptosis. Therefore NOD/SCIDγc^null^ mice were injected intra-tibially with luciferase-tagged myeloma cells. Disease progression was monitored by weekly bioluminescent imaging (BLI) and measurement of paraprotein levels. Results were compared with magnetic resonance imaging (MRI) and histology. Assessment of model suitability for preclinical drug testing was investigated using bortezomib, melphalan and two novel agents. Cells engrafted at week 3, with a significant increase in BLI radiance occurring between weeks 5 and 7. This was accompanied by an increase in paraprotein secretion, MRI-derived tumour volume and CD138 positive cells within the bone marrow. Treatment with known anti-myeloma agents or novel agents significantly attenuated the increase in all disease markers. In addition, intra-tibial implantation of primary patient plasma cells resulted in development of myeloma within bone marrow. In conclusion, using both myeloma cell lines and primary patient cells, we have developed a model which recapitulates human myeloma by ensuring the key interaction of tumour cells with the microenvironment.

## Introduction

Multiple myeloma is caused by clonal expansion of malignant plasma cells within the bone marrow. Acquired genetic mutations within myeloma cells and their interaction with bone marrow stromal cells (BMSCs) contribute to disease progression and drug resistance [1]. Myeloma cells and BMSCs quickly become mutually dependent on one another through a set of feedback loops comprised of various secreted cytokines and growth factors. This leads to dysregulation of key processes contributing to enhanced drug resistance.

An animal model that accurately reflects human myeloma and takes into account the protective nature of BMSCs would be powerful in confirming the efficacy of therapeutic agents *in vivo*, and accelerate the drug development process. A number of different animal models are currently used to study myeloma, including 5TMM, together with various SCID xenografts, and transgenic models [2]–[11]. Although these models have been useful in highlighting the importance of certain genetic events and signalling pathways in myeloma, they have a number of limitations. These include the use of mouse, rather than human, myeloma cells, a lack of tumour homing and interaction within the bone marrow, the presence of extramedullary disease particularly in the lungs, spleen and liver (areas not affected in patients), and a long latency period before disease establishment.

We believe the ideal model to test novel agents in a preclinical setting should possess the following characteristics; 1) the disease should be representative of the human condition, 2) there should be a relatively quick time to progression from initial inoculation, and 3) there should be markers to monitor the disease during treatment.

In order to address the limitations of these previous models, we report an adapted version of a model set up in AML [12] for the study of myeloma development and treatment. In this model we have shown successful engraftment of human myeloma cell lines and primary human material directly into the bone marrow of mice. The progression of the resulting tumours were tracked using bioluminescent imaging (BLI) and validated with a series of techniques including serum paraprotein measurement (ELISA), magnetic resonance imaging (MRI), flow cytometric analysis of CD138 expression and histology. Furthermore, we show that this model is suitable for the assessment of response to therapy with drugs known to be efficacious against myeloma, bortezomib (BZB), and melphalan, and two novel agents, an aminopeptidase inhibitor and a HDAC inhibitor which we have previously shown to be effective *in vitro*, and which has recently shown efficacy in a phase I/II trial in acute myeloid leukaemia and myeloma [13], [14].

## Materials and Methods

Procedures involving animals were approved by the Home Office and carried out within guidelines set out by both the Institute of Cancer Research’s Animal Ethics Committee and national guidelines according to the United Kingdom Coordinating Committee on Cancer Research [15].

### Cell Lines, Patient Cells and Reagents

U266 myeloma cells (ATCC) were transfected pcDNA3.1^luciferase^ (a kind gift from Gary Box, ICR) using Amaxa® Nucleofector® Kit C (Lonza), according to the manufacturer’s protocol. Following nucleofection, cells were cultured in RPMI-1640 GlutaMAX™ medium (Invitrogen) (supplemented with 10% heat inactivated foetal calf serum, 60 µg/ml penicillin, and 100 µg/ml streptomycin) and G418 (Invitrogen; 2 mg/ml). Tosedostat and CHR-3996 were acquired from Chroma Therapeutics Ltd. Stock solutions were made in 100% DMSO and stored at -20°C. Working solutions were made by dilution with phosphate buffered saline (PBS; Invitrogen). Bortezomib (BZB) was obtained from Johnson & Johnson and reconstituted in 0.9% w/v saline. Once reconstituted, BZB was administered within 2–3 hours and discarded afterward. Melphalan (Sigma) was dissolved in ethanoic acid and diluted with PBS for administration.

### Intra-tibial inoculation of tumour cells

Female NOD/SCIDγc^null^ mice (The Jackson Laboratory, USA) approximately 6 weeks old were anaesthetized using Hypnorm/Hypnovel/water in a ratio of 1∶1∶3. Mice were inoculated via intra-osseus injection in the tibia with either 1×10^5^ or 2×10^6^ U266^luciferase^ myeloma cells in 20 µL RPMI-1640. Mice were monitored for myeloma progression over 8–10 weeks by weekly bioluminescent imaging (BLI) and paraprotein level measurement.

Peripheral blood was taken from 3 plasma cell leukaemia patients and plasma cells were extracted by Ficoll-Paque. To assess the engraftment of patient cells, mice were inoculated with either 1×10^5^, 1×10^6^ or 1×10^7^ cells in 20 µL complete RPMI-1640 GlutaMAX™ medium. Mice were monitored for myeloma over 5 months. Post-mortem flow cytometry was used to assess tumour infiltration.

### Treatment schedules

Mice injected with U266^luciferase^ cells were assigned into the following three treatment groups: no treatment control (n = 6), 0.8 mg/kg BZB I.P. (n = 9), and 75 mg/kg tosedostat I.P. (n = 9). In addition a negative control group of mice that were not injected with cells and did not receive treatment was included. Treatment commenced 5 weeks after tumour cell inoculation and continued for 4 weeks. Tosedostat treatment was maintained for 6 days out of 7 each week. BZB was administered twice weekly.

### *In vivo* bioluminescence imaging

Mice were imaged at weeks 4 and 9 post-inoculation in order to assess tumour burden pre- and post-therapy. Mice were anaesthetized using isoflurane and imaged 9 minutes after I.P. injection of D-luciferin (1.5 mg/mouse, Caliper Life Sciences). Using an IVIS^®^ Imaging System 200 Series (Xenogen), images were acquired by 60 second exposure. Luciferase signal intensity was quantified by measuring average radiance (p/s/cm^2^/sr) using Living Image 4.0 software.

### Measurement of serum paraprotein levels

Serum samples were acquired from each mouse by tailbleed for the measurement of human Igλ in murine serum using enzyme linked immunosorbent assay (Human Lambda ELISA Kit; Bethyl Laboratories Inc). The assay was carried out as per the manufacturer’s protocol.

### Magnetic Resonance Imaging (MRI)

MRI scans were acquired on a 7T horizontal bore Bruker system. Mice were anesthetized using Hypnorm/Hypnovel/water (1∶1∶3), restrained with dental paste to limit motion artefacts, and positioned supine within a 3 cm birdcage ^1^H coil. T_2_-weighted TurboRARE images were acquired using TE = 24.6 ms, TR = 6000 ms, coronal slice thickness 0.3 mm, FOV 30×30 mm, matrix 100×100, 16 averages, AQ ∼14 min. On these T_2_-weighted images, tumour was identified as a hyperintense signal enclosed within the cortical bone. Tumour burden was quantified from regions of interest drawn on the periphery of the hyperintense signal in OsiriX and followed through each slice, for each bone within both legs.

### Flow cytometry & histology

Bone and tissue samples were collected, homogenized and stained with anti-CD138-APC antibody (BD Biosciences) to identify the presence of human myeloma cells, according to the manufacturer’s protocol. Stained samples were then analyzed using an LSRII flow cytometer (BD Biosciences) and FACSDiva Software. Bones (tibia and femur) were excised for histological examination and fixed in neutral buffered formaldehyde (10%) overnight at 4°C. Samples were washed with water and decalcified in 10% EDTA (pH7.4) for 14 days, until they lost normal structural rigidity. The bones were then embedded in paraffin bocks and 5 µm sections cut. Sections were subsequently stained with haematoxylin and eosin (H&E), and immunohistochemically processed using anti-CD138 antibodies, and evaluated by histopathology. Sections were visualized and recorded on a BX51 microscope (Olympus Optical Co. Ltd, UK) equipped with a driveable stage (Prior Scientific Instruments, UK), using CellP software (Soft Imaging System, Germany).

### Statistical analysis

All data are presented as mean ± SEM. Statistical analysis was conducted using either 1-way or 2-way ANOVA with Bonferroni post-test, unless stated otherwise in the text. GraphPad Prism 5.01 software was used for these analyses.

## Results

### Optimization of cell number

Initial experiments were conducted in order to optimize the cell number for implantation. Time to hind limb paralysis and Igλ levels were monitored. Mice were injected intra-tibially with either 1×10^5^ (cohort A) or 2×10^6^ (cohort B) U266^luciferase^ cells. Over the next 8 weeks, blood samples were collected in order to examine serum levels of human Igλ. Paraprotein levels in cohort A did not increase until week 5, compared to week 3 in cohort B. By week 8, cohort B had high levels of Igλ at 2998.7 ± 247.6 ng/ml, compared to 1278.3 ± 20.9 ng/ml in cohort A (Figure S1). In both cohorts, post-mortem examination revealed severe spinal deformity plus no organ involvement, both representative of human myeloma pathogenesis and progression. From these results it was decided that 2×10^6^ cells would be used to inoculate animals given the shortened lag time to development of myeloma.

### Engraftment and growth of U266^luciferase^ cells in NOD/SCID-γ IL2R^-/-^ mice monitored by bioluminescence imaging (BLI)

**Figure 1:**
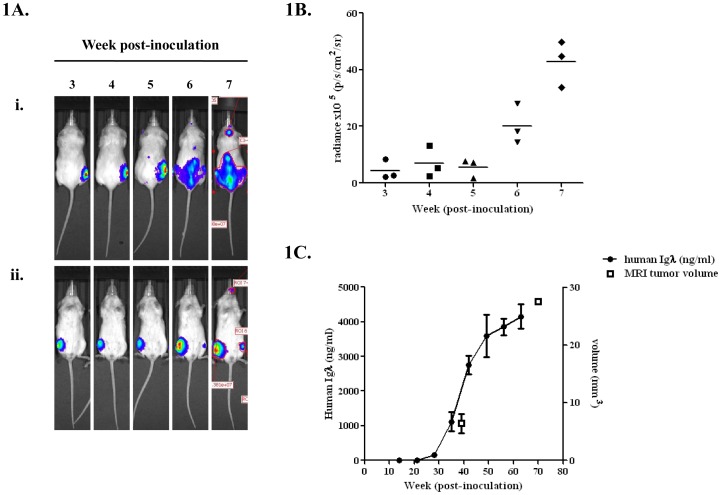
Engraftment and growth of U266^luciferase^ cells as monitored by BLI, serum paraprotein and MRI. A. (i) Dorsal and (ii) ventral BLI acquired from IVIS over weeks 3–7 post-inoculation. **B.** Quantitative measurement of radiance from BLI. Radiance reflects the intensity of luciferase luminescence and therefore number of luciferase-tagged cells present. Results show that radiance increases in a time-dependent manner over the course of the experiment and that a significant increase in radiance occurs over weeks 5–7 (p<0.05, 1-way ANOVA with Bonferroni post-test). **C.** Paraprotein levels in the serum increases in a time dependent manner and correlates with the increase seen in BLI. MRI-derived tumour volumes determined at approximately weeks 5 and 10 confirmed tumour progression seen with BLI.

Engraftment of U266^luciferase^ was first observed approximately 3 weeks post-inoculation (Fig 1A). BLI acquired at weeks 6–8 revealed spread to the pelvis, contralateral femur, tibia and spine. The endpoint of the experiment was reached at weeks 8–9 when animals developed hind limb paralysis. Quantitative BLI measurements were used to construct growth curves which corresponded to tumour load (Fig 1B). BLI showed a significant increase in radiance from 5.6×10^5^ to 43.0×10^5^ p/s/cm^2^/sr between weeks 5 and 7 (p<0.001, 2-way ANOVA with Bonferroni post-test).

### Validation of bioluminescence imaging: serum paraprotein levels and MRI

Serum paraprotein levels were measured by ELISA in parallel with BLI (Fig 1C). In correlation with initial engraftment at week 3, there was a corresponding increase in Igλ levels. Serum levels of Igλ increased from 149.3 to 4152.1 ng/ml during weeks 3–9 (p<0.05). MRI images acquired at weeks 4 and 8 showed a significant increase in intra-bone signal intensity, from 6.4 to 27.6 mm^3^ (p<0.05, Student’s paired t-test). These data confirm the changes seen in BLI.

### Validation of bioluminescence imaging: CD138 expression & histology

**Figure 2:**
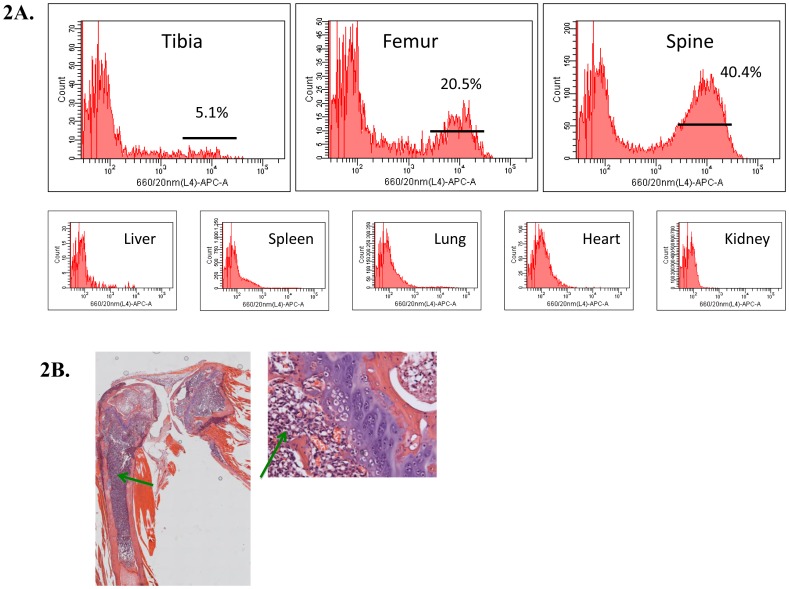
CD138 expression and histology validate BLI, paraprotein and MRI results. A. Flow cytometry histograms show the presence of CD138^+^ cells in the tibia, femur and spine of myeloma mice. Cytometric evaluation of the organs confirmed that the cells were confined to the bone marrow. **B.** Histological analysis of the tibia and femur confirmed the presence of a high number of CD138^+^ plasma cells (green arrows), resulting in the loss of classical bone marrow architecture.

Analysis by flow cytometry, post-mortem, confirmed the presence of CD138 expressing human myeloma cells in the bone environment and the absence of CD138 expression from all organs (Fig 2A). Following injection of cells into the tibia, tumour cells were seen in the femur, contralateral tibia and femur as well as the spine. This was confirmed by histological samples. Fig 2B shows the staining of U266^luciferase^ cells with haematoxylin/eosin, in the tibia and femur. Results from histology correlated with the results from flow cytometry. Results from this pilot study confirm that injection of human myeloma cells directly into the bone of NOD/SCIDγc^null^ mice results in disease characteristics similar to those observed in patients with an increase in paraprotein levels, disease confined to the bone marrow, spinal progression and eventual paralysis.

### Adaptation of model for primary patient material

In order to determine whether the direct delivery of tumour cells to the bone marrow could also result in the growth of patient primary cells, 10 mice received intra-tibial inoculation of material from 3 cases of plasma cell leukaemia with complex cytogenetics. Mice were inoculated with either 1×10^5^, 1×10^6^ or 1×10^7^ CD138^+^ cells in 20 µL complete RPMI-1640 GlutaMAX™ medium and monitored for signs of myeloma development, including hind limb paralysis, over a period of 6 months. Mobility issues developed in 6 mice injected with 1×10^6^ and 1×10^7^ cells, from all 3 donors (2 with additional plasmacytoma localized at the injection site). Flow cytometry demonstrated confinement to the bone marrow (Figure S2). Even with small numbers, a correlation between lag time and cell dose was apparent (data not shown). The ability of the mice models to support the growth of patient cells is unusual and highlights the importance of the direct intra-tibial injection route in allowing the myeloma cell:BMSC interaction.

### The intra-tibial model of myeloma bone disease can be used to monitor drug response

**Figure 3:**
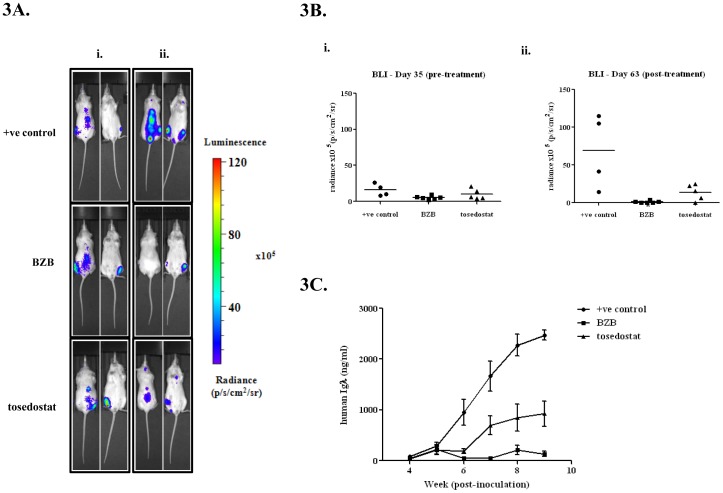
BLI and serum paraprotein changes in response to therapy. **A.** (i) Pre-treatment and (ii) post-treatment BLI of mice at weeks 5 and 9. **B.** Quantitative measurement of radiance from BLI. (i) No significant difference in radiance between treatment groups was seen at the start of the treatment schedule. (ii) Post-treatment radiance levels revealed a significant attenuation of tumour spread by both BZB and tosedostat (p<0.05, 1-way ANOVA with Bonferroni post-test). **C.** Paraprotein levels during treatment schedule. Positive control mice showed an exponential increase in serum levels of Igλ over 9 weeks. In comparison, both treatment groups did not exhibit the same increase, with significantly lower levels by the end of treatment (p<0.05).

Having demonstrated the development of a suitable preclinical mouse model and strategy to track the progression of myeloma, we then investigated its suitability in testing anti-myeloma therapies, specifically the effective myeloma therapeutic, bortezomib (BZB), and a novel aminopeptidase inhibitor, tosedostat. Tosedostat has previously been shown to inhibit cell proliferation, induce cell cycle arrest and apoptosis in myeloma cells *in vitro*.^13^ Therapy was divided into 3 groups; BZB treated, tosedostat treated and positive control. An additional group of non-inoculated mice was used as a negative control. At the beginning of the treatment schedule (Week 5), each group of mice displayed similar BLI radiance and paraprotein levels (**Fig 3A & 3B**). At the end of the 4 week treatment schedule, BLI was performed again. In the control group, there was a significant increase in radiance from 16.1 ± 4.2×10^5^ p/s/cm^2^/sr to 69.0 ± 24.4×10^5^ p/s/cm^2^/sr (p = 0.01 students t-test). In comparison, in the BZB and tosedostat treatment groups, no significant increase in radiance was seen (BZB: 5.2 ± 1.1×10^5^ p/s/cm^2^/sr to 1.1 ± 0.6×10^5^ p/s/cm^2^/sr; tosedostat: 9.9 ± 3.2×10^5^ p/s/cm^2^/sr to 13.8 ± 4.7×10^5^ p/s/cm^2^/sr). This effect was also seen using another standard anti-myeloma agent, melphalan and a novel histone deacetylase inhibitor CHR-3996 (Figure S3). BLI imaging of negative control mice did not reveal luciferase activity.

Paraprotein levels mimicked these changes in BLI (Fig 3C). Serum Igλ levels diverged at week 6 with BZB mice displaying the lowest level at 49.0 ± 19.6 ng/ml, which was significantly different from control, at 948.4 ± 255.9 ng/ml (p<0.01, 2-way ANOVA with Bonferroni post-test). Tosedostat treated mice also had significantly lower Igλ levels than control, at 191.0 ± 46.9 ng/ml (p<0.01, 2-way ANOVA with Bonferroni post-test). By the end of treatment, Igλ levels in control, BZB and tosedostat treated mice were 2473.7 ± 211.7, 132.5 ± 50.8 and 923.0 ± 248.6 ng/ml, respectively. Igλ levels in both treatment groups were highly significantly different from control (p<0.001, 2-way ANOVA with Bonferroni post-test). Again this effect was also seen using melphalan and CHR-3996 (Figure S3).

**Figure 4:**
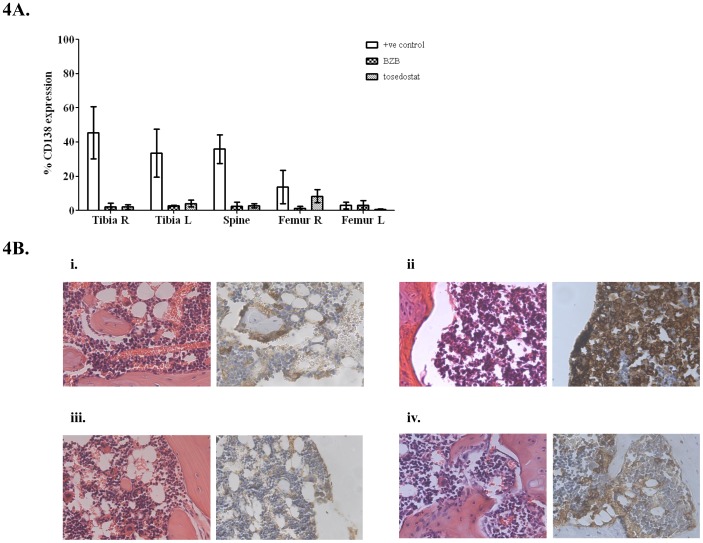
CD138 expression changes in response to therapy. **A.** Percentage of CD138^+^ human myeloma cells measured by flow cytometry in bone aspirates of mice (n = 3), showing a significantly lower percentage of positive cells in both tibias and spine of mice in the two treatment groups than in untreated mice (p<0.05, 2-way ANOVA with Bonferroni post-test). No CD138^+^ cells were observed in the organs of any of the mice. **B.** Histological analysis of sections from the tibias of mice from each group showed distinct differences. (i) Sections from healthy mice displayed classical architecture, with no CD138^+^ cells. (ii) In comparison, sections from untreated myeloma mice showed a high infiltration of CD138^+^ cells with loss of normal architecture. (iii) Treatment of mice with BZB resulted in the return of normal architecture and loss of CD138^+^ cells. (iv) A similar result was observed in mice treated with tosedostat, but with occasional scattered CD138^+^ cells.

Flow cytometry showed a significant reduction in CD138^+^ human myeloma cells in both tibias and spine in BZB and tosedostat treated mice compared to positive control mice (p<0.001) indicating decreased myeloma cell number (Fig 4A). Histological staining of paraffin embedded samples confirmed the observation made by flow cytometry (Fig 4B).

**Figure 5:**
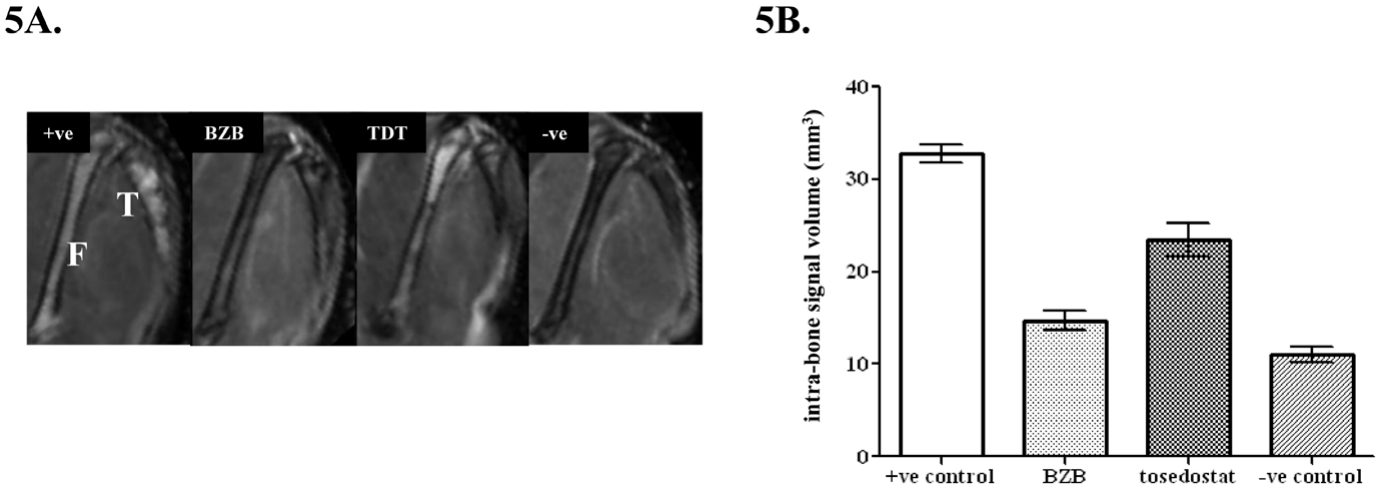
MRI changes in response to therapy. MRI-derived tumour volumes. **A.** Tumour was identified as a hyperintense signal enclosed within the cortical bone on T_2_-weighted images. MRI images showed a reduction in signal intensity in both treatment groups compared to positive control in both the tibia (T) and femur (F). **B.** Tumour volume was quantified from regions of interest drawn on the periphery of the hyperintense signal. Data are mean ± SEM, n≥6. Both BZB and tosedostat (TDT) treatment resulted in a significantly lower tumour volume compared to control (p<0.05, 1-way ANOVA with Bonferroni post-test). In addition, there was no significant difference in tumour volume between the BZB treated and negative control group.

MRI was used to determine the intra-bone tumour volume in each treatment group. Average tumour volumes were as follows: BZB group 14.7 ± 1.0 mm^3^, tosedostat group 23.5 ± 1.8 mm^3^ and positive control group 32.8 ± 0.9 mm^3^. Tumour volume for both treatment groups was significantly different from positive control (p<0.001, 1-way ANOVA with Bonferroni post-test). In addition, tumour volume for the bortezomib treated group was not significantly different from negative control mice (11.1 ± 0.8 mm^3^). (Fig 5). Furthermore, MRI images of the tibia and femur clearly demonstrate that BZB treatment significantly reduced the signal intensity compared to control and to a greater degree than tosedostat. The results gained from MRI correlate well with Igλ paraprotein levels, BLI, CD138 expression and histology.

These data show that this model is clinically representative of human myeloma development and progression, has a short latency to development and possesses various chemo-sensitive markers that can be used to monitor response to therapeutics i.e. MRI, BLI, paraprotein and the presence of CD138^+^ myeloma cells. These characteristics fulfil the criteria specified above for an ideal *in vivo* model of myeloma suitable for drug evaluation.

## Discussion

The development of a representative model of human myeloma for preclinical assessment of novel therapeutic agents is an important aim. In this study we present a novel, reproducible model of human myeloma that fulfils these criteria. The model was established by intra-tibial implantation of luciferase transduced U266 myeloma cells, into NOD/SCIDγc^null^ mice, and was confirmed using primary patient cells. The resulting tumours exhibited phenotypic, dissemination and growth patterns highly similar to those of human myeloma. Tumour engraftment was observed 3 weeks post-inoculation by BLI, with concomitant elevation of serum Igλ levels. The presence of tumour cells in the bone marrow environment was confirmed by examination of CD138 expression, MRI and histology. We then sought to evaluate its suitability for preclinical testing of novel myeloma therapeutics. Bortezomib and melphalan were used due their clinical success as therapy for myeloma. In addition tosedostat, a novel aminopeptidase inhibitor and CHR-3996, a HDAC inhibitor which have previously shown efficacy against myeloma cells *in vitro*. All therapies caused reductions in tumour load and associated myeloma markers.

There are a number of reasons why this model is superior to existing models and, therefore, provides an appropriate setting for therapeutic testing. A summary of existing *in vivo* models of myeloma suitable for drug evaluation can be found in **Table 1**. The 5TMM model was developed in 1979 after it was found that aging C57BL/KalwRij mice spontaneously developed myeloma [2]. Serial transplants using these murine myeloma cells were then conducted to create a syngeneic myeloma model. Recipients exhibit localization of myeloma cells to the bone marrow, increased paraprotein secretion and bone disease. Despite these features being common between murine and human myeloma, this model is limited by being solely murine-derived, and representative of only one type of myeloma. The NOD/SCIDγc^null^ model described herein is based on the implantation of human myeloma cells, which allows for different human myeloma subtypes to be investigated e.g. myeloma cells possessing different genetic backgrounds or with inherent resistance to a specific therapy. Additionally, engraftment of human cells is more relevant for investigating therapeutic targets and preclinical therapeutic testing, than murine myeloma cells.

**Table 1:** Summary of current mouse models for studying the development and treatment of myeloma.

Myeloma models	Description	Disadvantages
*5T series*	Intravenous injection of cells from bone marrow of C57Bl/KaLwRij mice showing spontaneous development of myeloma	System is entirely murine and genetic cause of myeloma development is unknown, therefore may not be similar to human disease.
*Chemically induced*	Intraperitoneal injection of pristane oil induces cancerous proliferation of plasma cells in the peritoneal cavity.	Lack of the following: tumor homing to the bone marrow, dependence on the bone marrow microenvironment and generation of osteolytic bone lesions. Therefore not characteristic of human disease pattern.
*SCID-hu/rab*	Myeloma cell lines/primary cells grown in irradiated human or rabbit fetal bone chip, which is then implanted into the mouse flank.	Ethically problematic. Fetal bone is stem cell rich, contains a high concentration of osteoblast precursor cells, and is isolated from general mouse physiology.
*Inoculation of SCID mice with human myeloma cells*	Intravenous or subcutaneous implantation of cells into murine host.	Lacks dependence on bone marrow microenvironment and frequently exhibits extramedullary involvement. Model also requires whole body irradiation pre-injection.
*Transgenic models*	Vk*myc	Does not exhibit all the important characteristic human myeloma cytogenetic events, limited to murine malignant cells in murine environment. Long latency to tumor development.
	XBP-1	
	c-MAF	
*Intra-tibial inoculation*	Cells directly implanted into host bone marrow.	Recapitulates human disease with myeloma confined to the bone marrow and disease response to therapy, easily measurable using BLI, paraprotein, MRI and histology.

The plasmacytoma model [3] produced by the injection of pristane oil into the peritoneum of Balb/c mice also has a number of limitations. Unlike myeloma, chemically induced plasmacytomas remain localized to the site of injection, do not produce bone lesions and do not rely on the host bone marrow microenvironment [16]. Furthermore, this model has a significant lag time (approx 120 days to development of ascites), and even then has variable incidence (∼60%).

Xenograft models are a popular choice for recreating myeloma in mice due to the speed of production and efficiency of engraftment. In subcutaneous xenograft models, cells are mixed with matrigel and injected into mice to form palpable tumours [17]. This route of administration means that tumours are localized and do not resemble the pattern of growth seen in human myeloma. In addition, this model does not take into account interactions with the bone marrow microenvironment, which are vital for proliferation, survival and drug resistance. Intravenous inoculation of myeloma cells into SCID mice has been exploited, but results in the widespread dissemination of myeloma to organs [18], [19]. Extramedullary involvement is a clinical characteristic of advanced myeloma but is not seen in early developmental stages. This suggests that tumours produced by intravenous and subcutaneous models do not rely on the bone marrow microenvironment. Additionally, both routes require whole body irradiation of mice prior to implantation. Two further models, the SCID-hu and SCID-Rab, are based on the implantation of either a human or rabbit foetal bone chip subcutaneously, into which myeloma cells are inoculated [19]. Within this environment, myeloma cells are able to proliferate and expand. However, this expansion is confined to the bone chip, and does not produce the pattern of spread and growth seen in human myeloma [4]. These features mean that pathophysiological symptoms such as hind limb paralysis cannot be assessed. In comparison to current xenograft models, the NOD/SCIDγc^null^ model described in the current paper is based on the direct implantation of myeloma cells into the bone marrow. The key advantages of this model are 1) these mice do not require whole body irradiation prior to tumour cell implantation 2) there is widespread skeletal involvement from a single inoculation site 3) there is no evidence of extramedullary involvement as shown by CD138 staining and histology 4) results are highly reproducible 5) the model culminates in an observable clinical endpoint i.e. hind limb paralysis.

It is important to consider how such a system differs from the more complex transgenic myeloma models that have recently been developed. Vk*myc, XBP-1 and c-MAF transgenic mice have been shown to develop myeloma-like malignancy, with localization of disease to bone marrow plus osteolysis [5]–[8]. The benefits of transgenic mice over traditional xenograft models are that mice are immunocompetent allowing insights into the role of immune response. In addition therapeutic agents can be evaluated at different stages of tumour development [9]. While these advantages are desirable, there are notable disadvantages to this model. Firstly, the tumours are murine in origin. Secondly, from a technical aspect, transgenic models are complex to create and there is often a long lag time to tumour development [20]. In comparison, the model described in this paper only takes 8 weeks from engraftment to exhibit hind limb paralysis, the end point of myeloma.

Other model systems have also been developed for the investigation of myeloma cell homing which could also be used for drug evaluation [10], [11]. The SCID-synth-hu model uses polymeric subcutaneous scaffolds inoculated with human mesenchymal stromal cells (MSCs) with tumour cells implanted at an external site. This model is relevant as it tackles the interaction between tumour cells and human bone marrow milieu and can, therefore, be used to assess preclinical agents and their effect on tumour homing. However, it does not result in infiltration of tumour cells into the host skeleton and is largely localized and lacks the representative nature of disseminated disease.

The final criterion which an ideal *in vivo* model should fulfil is that there should be chemo-sensitive markers to monitor during treatment regimes. The introduction of bioluminescent tagging of tumour cells has allowed for high-sensitivity imaging of myelomatic lesions in cancer models [21]. By tagging the multiple myeloma cell line U266 with the luciferase reporter, we were able to carry out repeated full body imaging over a period of weeks, to indentify focal areas of luciferase activity, indicating tumour burden. This is desirable as it is non-invasive, does not involve radioactivity, and does not require lengthy imaging of animals under heavy anaesthetic. The main benefit of having the luciferase marker is the opportunity for repeated rapid screening of test subjects in order to monitor the development of myeloma, and response to treatment. Results from bioluminescent imaging can then be confirmed and reinforced by examining other chemo-sensitive markers and techniques such as CD138 expression, histology, MRI and paraprotein levels. Results from the current set of experiments showed that these are suitable markers in testing the *in vivo* response to known myeloma therapies and newer novel compounds.

In conclusion using both myeloma cell lines and primary patient cells, we have developed a model which recapitulates human myeloma with secretion of paraprotein, disease confined to the bone marrow, lytic bone lesions and spinal compression. In addition, we have demonstrated this model is suitable for assessing the efficacy of both standard and novel therapeutics *in vivo*, using a number of non-invasive tumour markers such as BLI and MRI.

## Supporting Information

Figure S1 **Optimization of cell number for implantation into NOD/SCIDγc^null^ mice.** A. Inoculation of 1×10^5^ U266^luciferase^ cells caused an increase in serum paraprotein at week 5 and reached 1278.3 ng/ml by week 8. B. Inoculation of 2×10^6^ U266^luciferase^ cells caused an increase in serum paraprotein at week 3, and reached 2998.7 ng/ml by week 8. Mice injected with the higher dose of cells also developed hind limb paralysis (clinical endpoint of study) at week 8 whereas those with the lower dose did not. On the basis of these results, it was decided to use 2×10^6^ cells for inoculation due to shortened time to disease progression.
(TIF)

Figure S2 **Adaptation of model for use with primary cells.** Flow cytometry histograms show the presence of CD138 positive cells in the bone marrow of mice inoculated with primary patient material from 3 cases of plasma cell leukemia.
(TIF)

Figure S3 **Response to additional anti-myeloma therapies.** Additional experiments demonstrated activity of other known and novel therapies in the intra-tibial model. BLI and paraprotein levels both show the efficacy of melphalan (A) and CHR-3996 (B). Melphalan was given IP weekly (3 mg/kg) for 3 weeks and mice were sacrificed at wk7-8 due to loss of condition from melphalan treatment. CHR-3996 was given PO 6 times per week (50 mg/kg) for 4 weeks.
(TIF)
