# Supplementary figures and images for: Characterization of a Novel Mouse Model of Multiple Myeloma and Its Use in Preclinical Therapeutic Assessment
Source: PLoS One. 2013 Feb 21;8(2):e57641. doi: 10.1371/journal.pone.0057641 (PMC3578800; doi:10.1371/journal.pone.0057641)

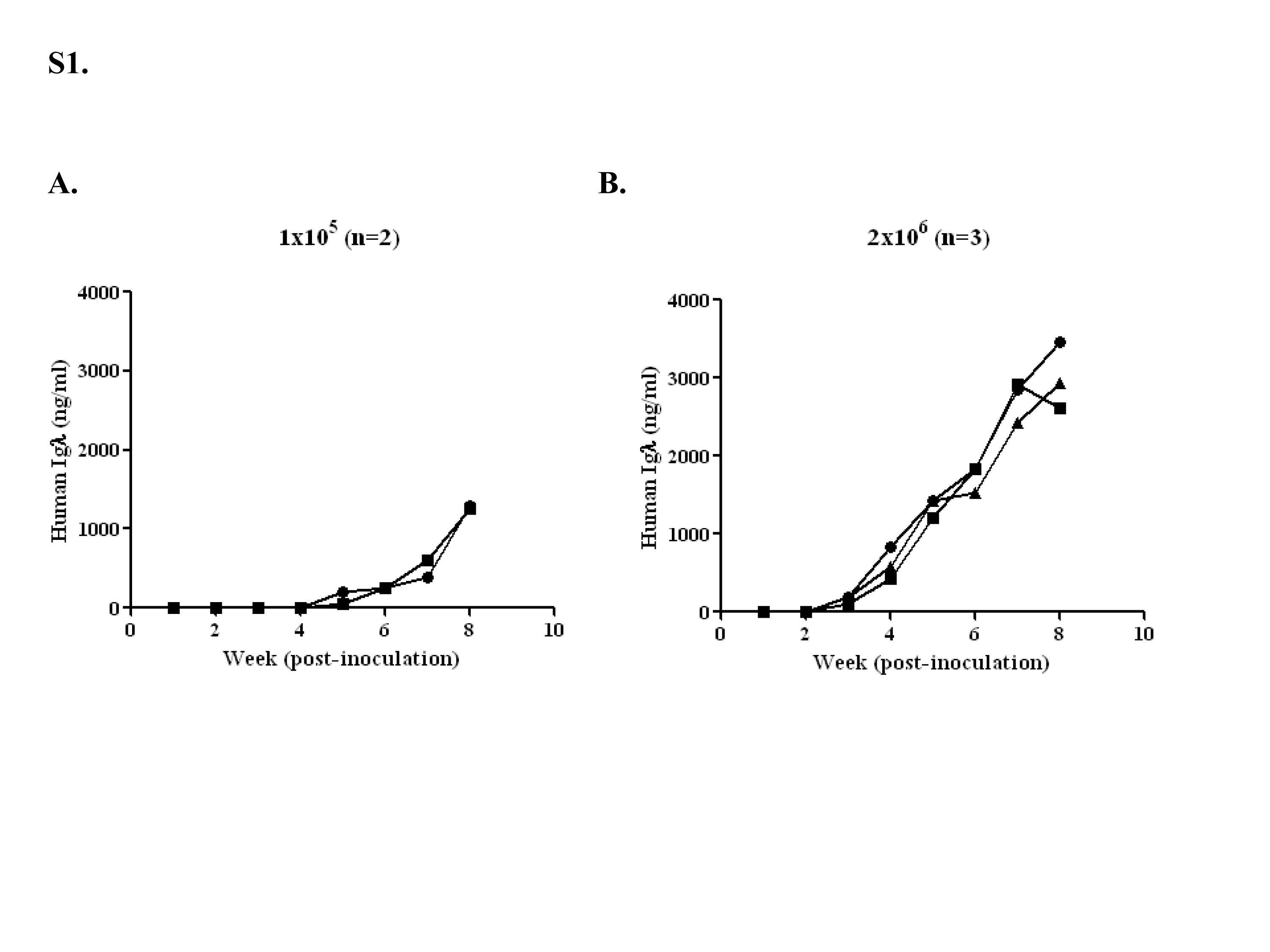

Supplement: Figure S1 — Optimization of cell number for implantation into NOD/SCIDγcnull mice. A. Inoculation of 1×10^5^ U266luciferase cells caused an increase in serum paraprotein at week 5 and reached 1278.3 ng/ml by week 8. B. Inoculation of 2×10^6^ U266luciferase cells caused an increase in serum paraprotein at week 3, and reached 2998.7 ng/ml by week 8. Mice injected with the higher dose of cells also developed hind limb paralysis (clinical endpoint of study) at week 8 whereas those with the lower dose did not. On the basis of these results, it was decided to use 2×10^6^ cells for inoculation due to shortened time to disease progression. (TIF) [file pone.0057641.s001.tif]

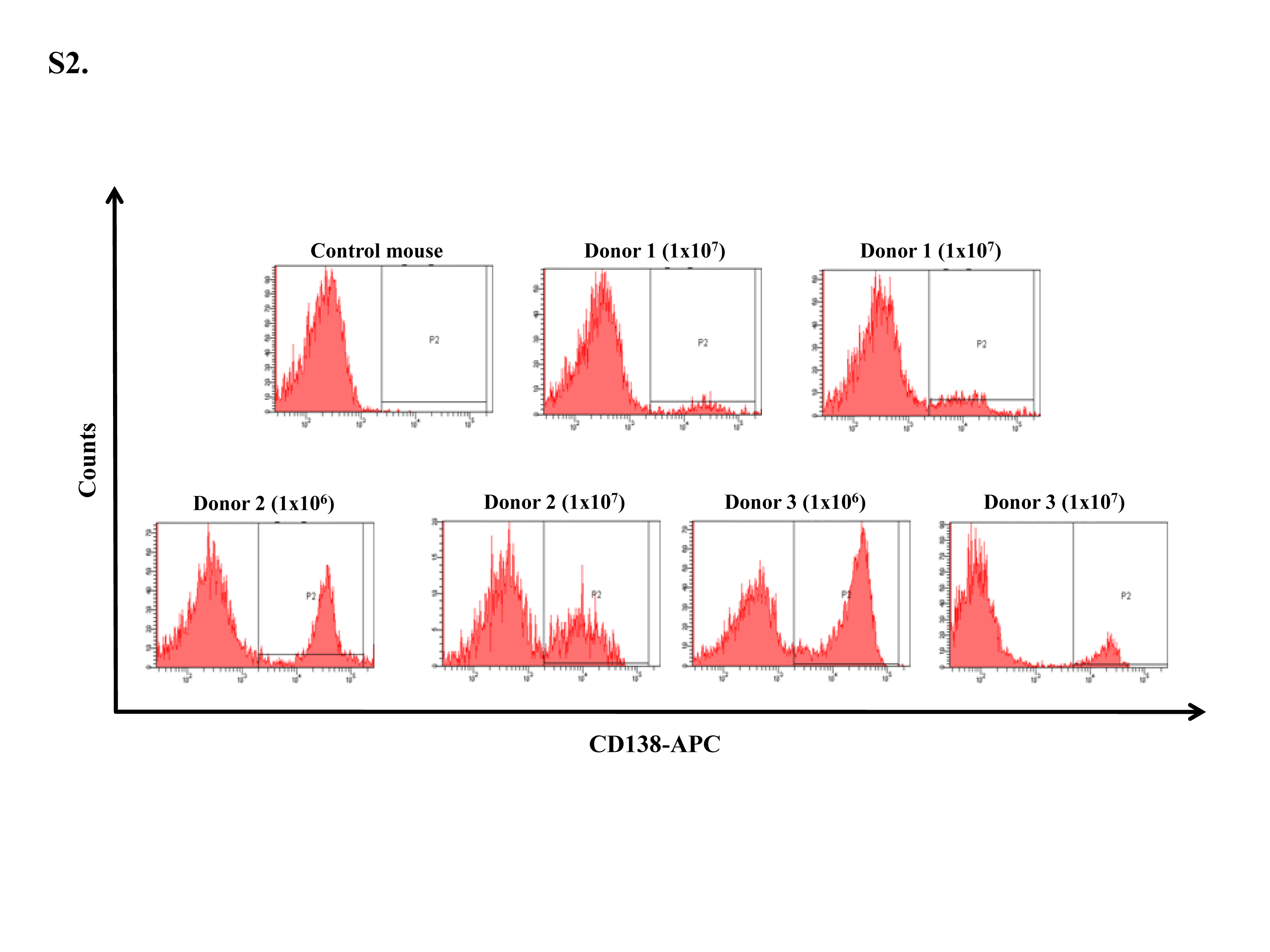

Supplement: Figure S2 — Adaptation of model for use with primary cells. Flow cytometry histograms show the presence of CD138 positive cells in the bone marrow of mice inoculated with primary patient material from 3 cases of plasma cell leukemia. (TIF) [file pone.0057641.s002.tif]

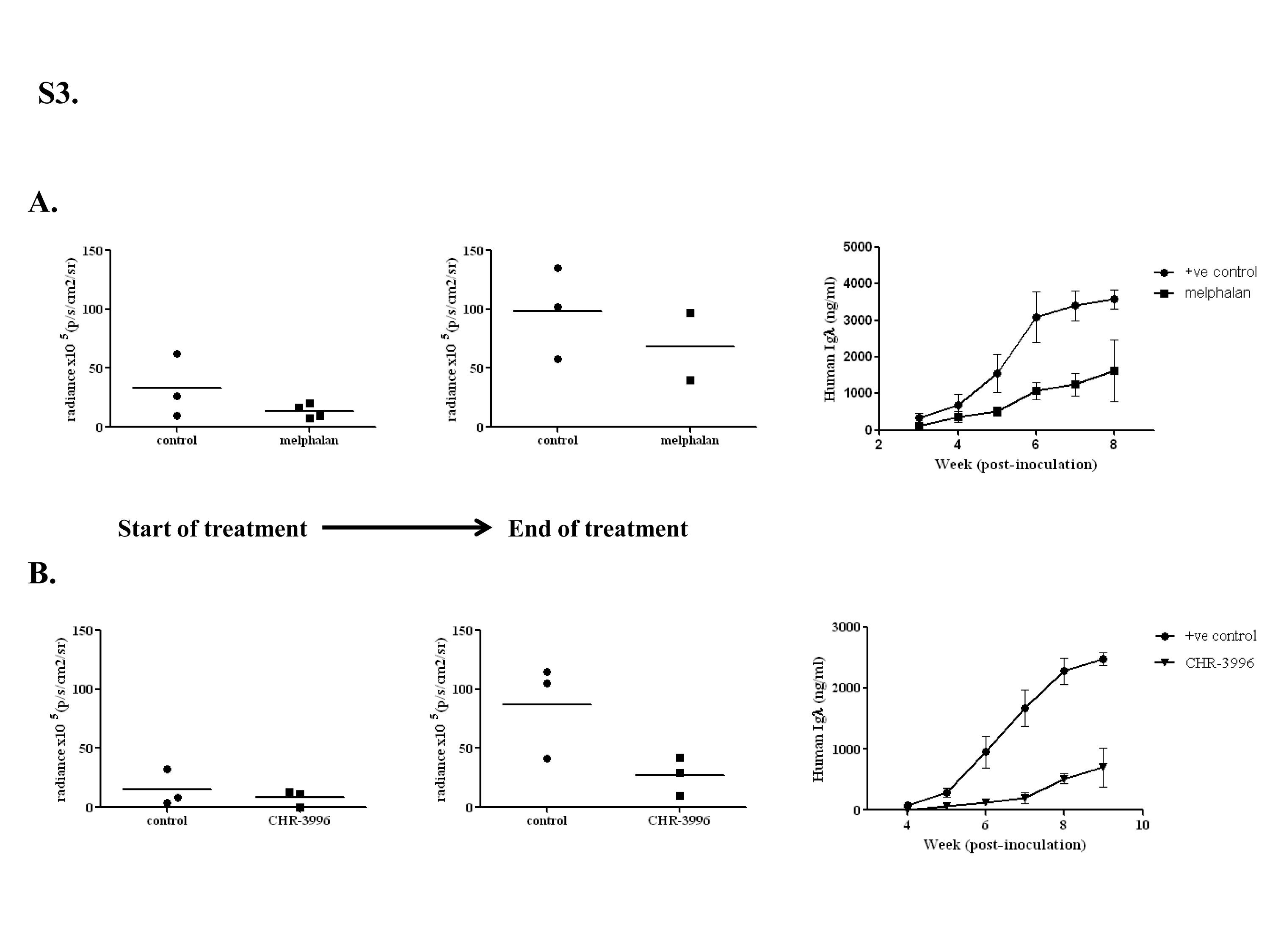

Supplement: Figure S3 — Response to additional anti-myeloma therapies. Additional experiments demonstrated activity of other known and novel therapies in the intra-tibial model. BLI and paraprotein levels both show the efficacy of melphalan (A) and CHR-3996 (B). Melphalan was given IP weekly (3 mg/kg) for 3 weeks and mice were sacrificed at wk7-8 due to loss of condition from melphalan treatment. CHR-3996 was given PO 6 times per week (50 mg/kg) for 4 weeks. (TIF) [file pone.0057641.s003.tif]
